# On Acute Articular Rheumatism

**Published:** 1873-06

**Authors:** Austin Flint

**Affiliations:** Professor of Practical Medicine, etc.


*On Acute Articular Rheumatism.* A lecture delivered at Bellevue Hospital, by AUSTIN FLINT, M.D., Professor of Practical Medicine, etc. (Reported phonographically for the Record).

GENTLEMEN:—My remarks to-day will be upon the subject of acute articular rheumatism, and they will be based upon the history of this man's case who is now before us. His name is P., æt. 22, Norwegian, and by occupation a blacksmith. To-day is the tenth day of his disease. I may here remark, that this disease, especially in its acute form, is not very frequently seen in this hospital. The present case does not show the disease in its acute form, for the patient is apparently approaching convalescence, yet it will serve to illustrate some of the important points pertaining to this disease. The word rheumatism is a term which has been used with a good deal of latitude, and under this name several different affections have been taken in, such as certain neuralgic troubles, certain affections of the muscles, and certain affections of the joints. When, however, we use the term articular rheumatism, we refer to a very distinct and clearly defined affection. It is a constitutional affection, characterized by local manifestations, consisting in an inflammation generally affecting several joints of the body, and not infrequently fibrous structures elsewhere than in the joints. It is an affection which usually attacks the greater part of the joints of the body, and these articular affections are but the local manifestations of the constitutional disease. These local affections are in certain points distinguishable from ordinary inflammation of analogous structures. We have no suppuration, none of the inflammatory products, no production of new tissue, which we might expect in ordinary inflammation, and these are essential points of difference which characterize these local manifestations of articular rheumatism. Another point which is very striking is, the inflammatory condition frequently subsides and disappears in a very short time; one day it is present, and the next day perhaps it may be entirely gone. The occurrence of these local manifestations in different joints successively is also a striking feature of the disease, as one joint may be affected to-day, and another to-morrow. Another feature which goes to show its constitutional character, and establish what has been said with regard to the local manifestations, is, the joints are affected symmetrically. The law of parallelism is strikingly illustrated in this disease; corresponding joints on both sides of the body are affected, and it is seldom that the strict law of symmetry is violated. If the joints upon both sides of the body are not affected, it does not violate the law, but it is simply the fact that the law is not illustrated. If corresponding joints are not affected, analogous ones are affected, such as the wrist and ankle perhaps, but an affection of the wrist and knee joint upon different sides of the body is rarely seen. This man is 22 years of age, which illustrates the fact that rheumatism, primarily, affects young subjects. A person, however, who has had the disease in early life, will be subject to repetitions of attacks ever afterwards, thus showing a constitutional tendency. Acute articular rheumatism also belongs among those diseases which are inherited, or rather, to which a predisposition may be transmitted. This man has generally had good health, and this is the first attack of the disease he has ever had. He was attacked in the day-time. It is more frequently the case that the patient is attacked in the night-time. The disease generally attacks suddenly, yet in a certain number of cases, the acute attack is preceded by more or less soreness and tenderness about the joints. As we look at the history of this case, we see that for two or three weeks this man had felt soreness in the joints, but not sufficient to prevent him from continuing in his occupation. The first manifestations which he had were in the smaller joints of the hands and feet; the carpal and metacarpal and tarsal and metatarsal joints. It first appeared in the right hand, then in the left foot; then in both knee-joints; then in both wrists; then in both shoulder-joints; and then in both hip-joints. The law of parallelism, it will be seen, was very well illustrated. At the present time the joints are comparatively but little affected. I deem it hardly necessary to dwell at any length upon the local symptoms, such as the pain and swelling which accompany the local manifestations; and will dismiss them with the simple remark, that we do not usually have much swelling and erythematous flush about the larger joints, such as the hips and shoulders. I must now ask you to mark the statement which I am about to make, as it is of special importance in connection with the topic I shall presently mention. The statement is this: this man has had no pain at any time on the chest, no præcordial pain at any time during the course of the disease. I will soon speak more particularly of the importance of this statement in the history of his case. This is a disease which in its acute form—for it is presented to us both as an acute and subacute affection—is characterized by a good deal of febrile movement pertaining to the disease itself, and also symptomatic. One of the popular names for this form of fever is "rheumatic fever," and it is not altogether improper, for the fever does not depend upon the local affection entirely, but it is sometimes altogether out of proportion to the local affection. We have, therefore, a fever which is partly essential and partly symptomatic. Last evening this man's pulse had fallen to 72 and the temperature in the axilla was 100¼. This affords a good illustration of the disparity which is sometimes seen between the pulse and temperature, as regards the criteria of a fever. When such a disparity is present, the temperature is entitled to the preference in deciding the question of fever or no fever.

This morning the pulse was 68. It is always to be borne in mind, that, in many diseases, about the time of convalescence, the patients have a pulse which is below the average frequency in the same persons in health. This is the case in typhoid and typhus fever, pneumonia, and some other affections. This morning there is no fever present, the temperature being 98¼.

This man has been taking quinine and nitro-muriatic acid. It might, at first, strike the mind as an incongruity in the treatment of this disease to administer an acid, while the predominant feature of the disease is the presence already of an acid in the system, but really there is no incongruity in it. Over and over again we apply remedies and measures which are directly antagonistical, and each will meet its own indication, and often the only proper method of treating certain cases is to meet the indications.

We come now to speak of one of the important events which is liable to occur in the course of inflammatory rheumatism. This disease in itself, as far as the constitutional difficulty is concerned, is not dangerous to life; but there is a danger in connection with certain incidental events, and those events which are most likely to occur relate to the heart. There is a special liability to an inflammation affecting one or both serous investments of this organ, and these are the untoward events to be looked after in cases of articular rheumatism. The importance of the disease and the permanent welfare relate chiefly to the occurrence of these complications. There are some other complications which are so infrequent that they do not give us much care, and we will pass their consideration. The cardiac affections are the prominent ones. Endocarditis occurs in quite a proportion of cases, but I do not give you the figures, because I think there has been some looseness and error in making up statistics upon this point. The reason for this

I will soon mention. Pericarditis is of much less frequent occurrence than endocarditis, and it may be said further with regard to these complications, that if we have pericarditis we have endocarditis, but the rule does not hold in the opposite direction. Pericarditis involves a certain amount of immediate danger, though a great proportion of cases get well. What we probably have in this case is endocarditis, and first of all we will study the evidence upon which this probability is based. The evidence in this case is not absolute, but it is *probable.* Now you will recollect the fact to which I called your special attention a few moments ago, viz., this patient has not had præcordial pain, or any chest symptoms whatever, during the progress of his case. The diagnosis of endocarditis is therefore based entirely upon physical evidence. This is the reason why endocarditis is a disease which has been discovered within the last half century, and was never before known. It was discovered by physical exploration, and must continue to be recognized by this means, because it occurs without any subjective symptoms. It is associated probably with some increase of the circulation, but as this increase goes more or less with the rheumatism, we cannot draw the inference from this that endocarditis is present. How are we to determine whether a patient has endocarditis or not, who is suffering with articular rheumatism? We are to reach a positive diagnosis in this way: if the patient be under your observation, and you can determine by auscultation that there is no mitral systolic murmur present at the commencement of the attack, and then in the course of the disease a mitral systolic murmur is developed, you know that the patient has endocarditis. It all depends upon the development of this mitral systolic murmur, and the murmur is the hinging point. This patient has a mitral systolic murmur, but the diagnosis is not positive, because the patient had the same murmur when he came into the hospital, and we do not know certainly that the murmur has been developed since the commencement of the disease. It has probably been developed in this patient since the commencement of this attack, for it is the first attack the patient has had of the rheumatism; he has always been well, and as the murmur is one which does not indicate regurgitation, it is altogether probable that in this case it is evidence of endocarditis. I find here that the apex of the heart is beating in the fourth intercostal space, as it not infrequently does when the body is in a recumbent position. By percussion I determine that the heart is not enlarged. This would not be the case if the patient had had mitral disease for any length of time previous to the present attack, for he would have more or less enlargement of the heart.

Within a certain circumscribed space about the apex of the heart, I get a murmur with the first sound of the heart, a mitral systolic murmur, and it is not propagated much beyond this quite limited area. It is not proper to call this murmur a mitral regurgitant murmur, because there is no evidence of regurgitation.

What do we look for as physical evidence to show that there is regurgitation? The fact that a mitral murmur is present, is not limited to the apex, is tolerably loud, and is propagated to the left, would be evidence that it was one of regurgitation.

I also get a murmur at the base of the heart, but I attach no special importance to this, because we cannot attach much importance to a murmur at the base of the heart in a case of articular rheumatism. It is very frequently present, and is dependent upon the condition of the blood. It is always present in females, or at least I believe I have never seen a case of articular rheumatism in a female where this murmur was not present. It is just here, I apprehend, that great confusion has arisen with regard to statistics in reference to endocarditis, and many cases have been called endocarditis in which the disease did not exist. I would not make my diagnosis relying upon this murmur at the base, unless I had the mitral systolic murmur at the same time.

Endocarditis is a serious complication, because in it the rheumatism has laid the foundation for the subsequent occurrence of valvular lesions.

We have attenuation of the valves, thickening and calcification of the valves and other valvular lesions, all arising from an endocarditis in connection with rheumatism, and we have not much knowledge of endocarditis except in connection with these cases of articular rheumatism. We come now to ask the question, what are the indications in the treatment of acute articular rheumatism? In the first place we would like to cut it completely short if possible, but we cannot do this often, if ever. Next, we would like to abridge its duration, and there is reason to believe that this may be done to a certain extent. The great object, however, is to prevent these cardiac complications, pericarditis and endocarditis, for if a patient passes through this disease and escapes these complications, he is exceedingly fortunate.

Just here, however, a knowledge of the natural history of the disease, based upon the observation of cases which have been permitted to run their course without the influence of therapeutical interference, is of service in making up our estimate of the value of treatment. In the year 1862, I conceived a plan of observing cases of articular rheumatism, without the use of medicine. The reason for so doing was because almost all the cases reported as having been under the influence of any special plan of treatment, such as mercurialization, colchicum, bleeding, blistering, etc., were reported as cured, and hence each plan of treatment was reported as being attended with the greatest success. I therefore reasoned that the probability was, inasmuch as all the different modes of treatment tended to success, that the disease itself tended towards getting well. I accordingly treated 13 cases in this hospital, and they got no remedy at all, except one which was intended for a moral effect upon the patients, and that, for the sake of giving it a name, was called the placeboic remedy. The only treatment which these patients received, aside from this placeboic remedy, was a little anodyne and local applications to the joints of a palliative character. I resolved to continue the plan of treatment until something occurred to render it improper to continue it longer, and in only one of the 13 cases treated after this method did any complication occur, and that patient had the complication when she came into the hospital. The average duration of the disease in those cases was 26 days, and no important complications took place. I reported these cases in an article entitled "A contribution toward the Natural History of Articular Rheumatism," which was published in the *American Journal of the Medical Sciences*, July, 1863. I hope I shall not be thought egotistical in referring to these observations. They were made in this hospital, and my object in making them was stated to the class then in attendance. So far as I know, a series of similar observations had never before been made. I am led to assert my claim to whatever credit may belong to precedence in this line of investigation, because shortly after my observations a similar plan for the same object was pursued by others. Guy's Hospital Reports, volume for 1865, contains a report of a considerable number of cases treated by Dr. Gull, chiefly with mint water; and another report of additional cases was made in an article by Dr. Gull and Dr. Sutton, contained in the "Transactions of the Royal Medical and Chirurgical Society of London," in 1869. I should not thus expose myself to the charge of egotism in asserting my claim to priority in the study of the natural history of articular rheumatism, had these observers made any reference to my article in the *American Journal of the Medical Sciences*. I feel bound to make this claim, not alone for myself, but for this hospital and for American clinical medicine.

Dr. Fuller, who is the author of the so-called "Alkaline treatment," states that cardiac complications will not arise after the alkalescence of the urine is once established, but I think this author is too ardent in his statements, for I have seen cases in which endocarditis has been developed while the patients were fully under the effect of the alkaline treatment. Statistics, however, show that there is a diminished *liability* to these complications, and therefore we are not warranted in repeating observations without remedies. The method of treatment to be pursued in a case of acute articular rheumatism, is the adoption of what is called the alkaline treatment. The prime object in this treatment is, to produce alkalinity of the urine, regarding that as the criterion that the system is sufficiently affected in as short time as possible, for we cannot tell at what instant the complications may appear.

To accomplish this, the bi-carbonate of soda or potassa may be given in half-drachm or drachm doses every two hours, and by these doses you can render the urine alkaline within twenty-four hours at the farthest, with a good deal of certainty. After the urine has been rendered alkaline, the remedy is to be continued in varying doses sufficient to maintain the urine in an alkaline condition during the continuance of the disease. In this case before us, the disease has continued only ten days, and the patient is apparently convalescent.

Quinia in full doses also forms a good adjuvant in the treatment, as the patient is becoming convalescent. It contributes very much to the welfare of the patient. The joints are to be treated by palliating applications. Frequently you will find that shampooing the joints, be they never so tender, is very beneficial, commencing with gentle frictions, and gradually increasing the force as the patient can bear. Fomentations which contain alkalies and anodynes may be used also as local applications. As a rule, it is one of the great objects of medical treatment to relieve pain, for pain interferes with sleep and wears out the vital forces of the patient. Those patients afflicted with articular rheumatism may have opium sufficient to allay all irritation from pain, and give them quiet and rest. A more minute detail of the pathology of this disease must be considered at another time.

NOTE.—At a subsequent clinical lecture, Dr. Flint presented a case of acute articular rheumatism during the course of which peri-endocarditis (developed when the urine was alkaline), chorea, and right hemiplegia from embolism occurred. Coincident with the occurrence of the hemiplegia, a basic systolic heart-murmur, which had previously existed, disappeared.—*Med. Record.*

---

***Ventilation and Warming.***

In a lecture on ventilation, lately delivered before the Franklin Institute, Mr. L. W. Leeds gives the following practical directions concerning provisions for ventilation and warming in the construction of buildings: First, never have long underground fresh-air ducts. Second, never allow a sewer, soil-pipe, foul-air flue, or smoke-flue, to come near the fresh-air supply-flue, for fear of some connection being made between them by carelessness or accident. Third, never heat a building exclusively by currents of warm air. Fourth, always put the heating-flues on the outside walls instead of on the inside walls. Fifth, endeavor strenuously to avoid the fresh-air chamber becoming a common receptacle for all the rubbish of a filthy cellar.

